# SHERPA: an image segmentation and outline feature extraction tool for diatoms and other objects

**DOI:** 10.1186/1471-2105-15-218

**Published:** 2014-06-25

**Authors:** Michael Kloster, Gerhard Kauer, Bánk Beszteri

**Affiliations:** 1 AWI: Alfred Wegener Institute, Helmholtz Centre for Polar and Marine Research, Am Handelshafen 12, 27570 Bremerhaven, Germany; 2 HSEL: University of Applied Sciences Emden/Leer, Constantiaplatz 4, 26723 Emden, Germany

**Keywords:** Diatom, Segmentation, Outline, Elliptic Fourier analysis, Shape descriptors, Morphometrics, Automated slide scanning

## Abstract

**Background:**

Light microscopic analysis of diatom frustules is widely used both in basic and applied research, notably taxonomy, morphometrics, water quality monitoring and paleo-environmental studies. In these applications, usually large numbers of frustules need to be identified and/or measured. Although there is a need for automation in these applications, and image processing and analysis methods supporting these tasks have previously been developed, they did not become widespread in diatom analysis. While methodological reports for a wide variety of methods for image segmentation, diatom identification and feature extraction are available, no single implementation combining a subset of these into a readily applicable workflow accessible to diatomists exists.

**Results:**

The newly developed tool SHERPA offers a versatile image processing workflow focused on the identification and measurement of object outlines, handling all steps from image segmentation over object identification to feature extraction, and providing interactive functions for reviewing and revising results. Special attention was given to ease of use, applicability to a broad range of data and problems, and supporting high throughput analyses with minimal manual intervention.

**Conclusions:**

Tested with several diatom datasets from different sources and of various compositions, SHERPA proved its ability to successfully analyze large amounts of diatom micrographs depicting a broad range of species. SHERPA is unique in combining the following features: application of multiple segmentation methods and selection of the one giving the best result for each individual object; identification of shapes of interest based on outline matching against a template library; quality scoring and ranking of resulting outlines supporting quick quality checking; extraction of a wide range of outline shape descriptors widely used in diatom studies and elsewhere; minimizing the need for, but enabling manual quality control and corrections. Although primarily developed for analyzing images of diatom valves originating from automated microscopy, SHERPA can also be useful for other object detection, segmentation and outline-based identification problems.

## Background

Diatoms are a group of photosynthetic protists producing uniquely ornamented and diversely shaped silicate shells [1]. They are present in all aquatic and wet habitats and, with an estimated 10^5^ species, they represent the most species rich algal group [2]. Diatom assemblage composition reflects the abiotic and biotic features of their respective habitats, and is widely used for making inferences about environmental conditions in water quality monitoring and paleontology [3]. Due to a combination of traditional and practical reasons, the most widely applied method for diatom investigations is based on light microscopic analysis of so called permanent slides, prepared using the silicate frustules after cleaning them of organic material [1].

Size and shape distributions of diatom populations are measured and analyzed in a number of different fields, including taxonomy [4-8], ecology [9-12], and paleontology [13-16]. In such studies, dozens to hundreds of specimens are routinely investigated from each of several slides, and measurements are usually performed by one of the following methods: 1) through an ocular micrometer directly on images seen in the microscope by the investigator [17]; 2) as manual (mostly, length) measurements on digital live images presented on a computer screen [4,16]; 3) as manual (again mostly, length) measurements on saved digital images using general purpose image analysis software [12]; 4) combination of manual measurements and measurements obtained by custom-developed macros or extensions of general purpose image analysis software like ImageJ [16] or Optimas [5,7].

There is a considerable methodological gap between these approaches and the sometimes rather sophisticated methods which have been applied to diatoms in the image analysis literature for instance in the project ADIAC [18], or by others including [19-21]. Much of the experience gained in diatom image analysis studies should in principle be transferable to diatom morphometrics and would have the potential to speed up the latter and make it more accurate and reproducible. However, these methods have remained practically inaccessible to diatomists due to a lack of publicly available and user friendly implementations of image processing and analysis methods suitable for diatom analyses. Most of the diatom image analysis literature does not explicitly state which software tool or framework was used for implementing the applied methodology. Although this practice reflects a focus upon algorithms and methods, as opposed to software, and is probably well suited for readers with their main area of expertise lying in computer science and image analysis, translating these methodological experiences into routinely practicable workflows has remained a challenge beyond the qualification of most, if not all, diatomists, as illustrated by the almost complete lack of reports on re-use of these methods beyond the groups which developed them. The only case known to us where implementations of individual algorithms have been made available publicly is represented by the small collection of MATLAB and C source code files available under [22]. However, even these only represent fragments of a practically applicable analysis workflow and are virtually inaccessible to most diatomists (at least to the overwhelming subset lacking familiarity with MATLAB/C programming).

Several of the individual algorithms tested and applied in diatom image analyses in the above cited works represent standard image analysis methods, with widely available implementations in general purpose image analysis software like ImageJ [23]. Thus, it could be argued that such software should also be perfectly suited for the needs of diatomists. However, in our experience, whereas for instance ImageJ can be useful for processing and analyzing individual diatom images or small collections thereof, building a workflow for high throughput work with it requires serious programming capabilities, a reason probably hindering the use of such software in diatom studies. For instance, a number of segmentation algorithms can successfully be applied to diatom valves, but it is often found that a different method works best for different objects, depending not only on valve structure (and thus, also taxonomy) but also upon minor details of how the object lies relative to the focal plane and to neighboring objects [18]. Whereas one can easily apply a handful different segmentation algorithms to an image in for instance ImageJ, deciding which one gives best results in a case-by-case manner can be challenging. Doing so programmatically to enable batch processing of large numbers of images with minimal manual interaction would go beyond the capabilities of most non-image-analysis-expert users of ImageJ. Since diatom images are notoriously difficult to segment due to the optical properties of the silicate shells (low contrast, strong halo around outline, huge structural and shape diversity), chaining together individual analysis steps to an automated workflow also requires some kind of quality control. Differentiating objects of interest (diatom frustules, or, in particular cases, frustules of a particular group of diatoms) from other objects found by segmentation methods (sediment particles, debris, non-target species) would also require considerable programming skills to implement in ImageJ.

The outline represents a rather information rich aspect of the morphological variability of diatom frustules, and its shape and size contains substantial taxonomic and life cycle related information especially in the case of pennate diatoms (even if it has to be noted that diatom identification at the species level is mostly impossible based on outline shape alone). The main approaches for quantitative characterization of outline shapes in diatom morphometrics have included the use of simple heuristic shape descriptors like rectangularity [5], ellipticity, compactness [18,24]; Legendre-polynomials ( [6] and the large body of literature cited therein); Fourier descriptors [18,25,26]; and landmarks and semi-landmarks [8,27-31]. Although further methods have been developed, some specifically for diatoms, notably the segment shape analysis approach [32] successfully applied in [7], these have not become widely used. General purpose morphometrics software [33,34] is available for landmark and semi-landmark digitization and analysis, but using such software, landmark points need to be digitized individually and manually, hindering high throughput analyses. For other types of outline descriptors, some software support is available (see e.g. examples for software tools capable of calculating elliptic Fourier coefficients under [34]), but again not as part of routinely applicable workflows supporting the analysis of large numbers of images.

With SHERPA presented in the present paper, we address these gaps and introduce an easy-to-use tool for segmenting and analyzing light microscopic images of diatom frustules, and for extracting a number of outline features useful for diatom morphometrics (but potentially in other fields as well). Our goals were to develop a tool that implements 1) a full image analysis workflow from image segmentation to outline feature extraction, specifically adapted to diatom images, but potentially useful for other objects where outline shape is informative; 2) multiple segmentation methods and an automated selection of the best result for each segmented object; 3) matching of object outlines against a set of template outlines to enable both taxonomically selective as well as broader analyses; 4) object scoring and ranking to support quality checking; 5) extraction of a wide range of outline shape descriptors for further analyses; 6) supporting processing of large batches of images by minimizing the need for manual interaction, but leaving the possibility for it in case it should be required, e.g. to correct outlines for diatom valves with minor overlaps with neighboring objects. Software implementing statistical and/or machine learning methods for exploration, analysis, and classification of large multivariate data sets is widely available both commercially and free of charge for users at a wide range of levels of computer fluency (ranging for instance, from the easy-to-use PAST [35] or JMP [36] to the more challenging, but also more versatile statistical analyses systems like R [37] or SPSS [38]). Accordingly, we decided to not include this functionality in our tool but rather generate output that can be loaded for downstream analyses into the user’s statistical tool of choice.

### Implementation

**Figure 1:**
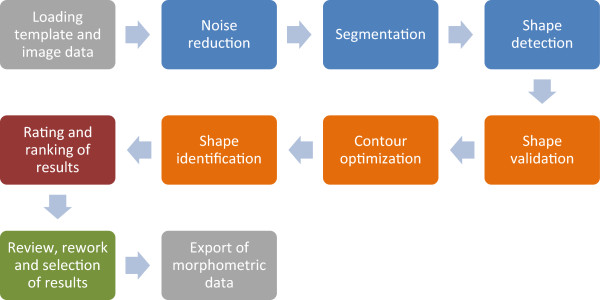
Structure of SHERPA‘s image processing pipeline/workflow.

SHERPA, the tool for "**SH**ap**E R**ecognition, **P**rocessing and **A**nalysis", offers an image processing workflow focused on the identification and measurement of object outlines (see Figure 1). Though it was developed focusing on analyzing diatom valves, SHERPA can also handle other object classes. Starting point are micrographs, obtained by optical microscopy, or similar images. For each depicted object, the respective outline is detected and compared to a set of templates which characterize representative shapes of interest. Detected objects receive quality scores and are ranked accordingly, reflecting the chance of representing a relevant object. The aim of this step is to reduce the effort required for sorting out unwanted objects. Suboptimal results can be revised manually to improve yield if necessary, and selected results can be exported along with a set of descriptors for further morphometric scrutiny.

This way, extensive image collections can be processed in a fully automated manner or with minimal manual intervention. Irrelevant data, originating from debris, damaged or unwanted objects, can be sorted out with little or no user intervention at all, while relevant objects are identified and measured. The exported morphometric descriptors allow for a detailed and specific analysis based on tools like R [37], and questions about variation in outline shape and size can easily be investigated.

One of the main strengths of SHERPA is its easily to follow workflow and plain user interface, which combine different techniques into a simple to use, yet powerful tool, which does not demand deeper expertise in image processing and programming. This distinguishes SHERPA from general purpose image analysis solutions like ImageJ [23], which usually require experience in image processing and a lot of manual intervention or skills in scripting (Table 1 lists the main features of SHERPA which go beyond those supported by ImageJ).

**Table 1:** Comparison of features of SHERPA and ImageJ

**Feature**	**SHERPA**	**ImageJ**
Integrated workflow for segmentation, identification and measurement of objects	Yes	No
Automatic combination of multiple segmentation methods	Yes	No
Automatic combination of multiple contour optimization methods	Yes	No
Convexity defect measures	Yes	No
Ranking of segmentation results	Yes	No
Quick interactive review of results	Yes	No

In order to create a low level entry point for novice users, extensive documentation is provided along with the software, including a comprehensive manual, a quick-start guide, a tutorial on how to achieve suitable settings in a straightforward way, and a technical description of the analysis process and extracted morphometric features.

SHERPA was developed for Windows7 64 Bit using C#/.NET 4.0. Most image processing functions are realized based on OpenCV 2.4.2 [39], whose DLLs are wrapped for .NET by Emgu CV 2.4.2 [40], and on ITK 4.2 [41] called via external executables. "Microsoft .NET Framework 4" [42] and the "Microsoft Visual C++ 2010 SP1 Redistributable Package (×64)" [43] have to be installed prior to running SHERPA. A 32 Bit version of SHERPA is available, but its usage is not recommended because it might run out of memory resources when analyzing large amounts of data.

#### Input data

*Image data* to be analyzed can depict objects either as dark structures on bright background (like obtained e.g. using bright field microscopy) or as bright structures on dark background (like obtained e.g. using dark field microscopy). Objects are identified by shape information. For proper results, object outlines should be focused as precisely as possible. Minor blurring will affect the accuracy of outline detection, while extensive fuzziness might impede usable results. For an optimal identification yield the sample density should be sparse without overlapping objects.

**Figure 2:**
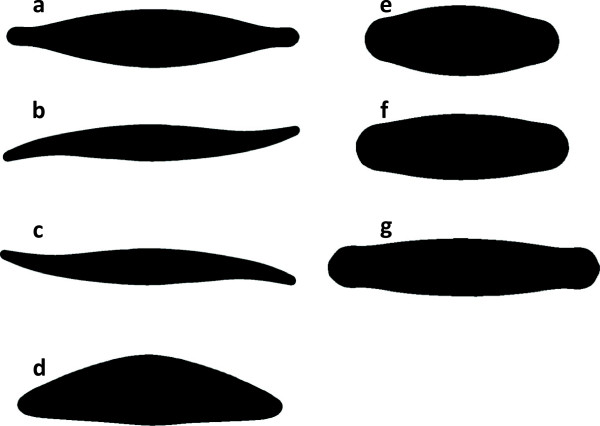
**Seven exemplary templates used for shape detection. a)** a typical *Navicula*, **b)** *Gyrosigma*, **c)** the same *Gyrosigma* mirrored, **d)** *Cymbella helvetica*, **e-g)** different variations of *Sellaphora pupula*. All shapes were derived from ADIAC data [44].

*Templates* provide prototypes of relevant shapes, containing silhouettes of each suitable object type (see some example diatom templates in Figure 2). A broad collection of templates depicting diatom valves is provided along with SHERPA (see under "Results and discussion"). However, for good results, a set of templates depicting the morphological variability of the objects under investigation must be generated. Depending on the object of interest, several templates might be needed to cover the range of shapes corresponding to one type (species). In the case of our objects of primary focus, diatom valves, templates should cover the range of shape variation occurring during size reduction for each taxon concerned (see some examples in Figure 2e-g).Since templates are matched to object shapes by using elliptic Fourier analysis (see below under "Shape identification"), the identification process is insensitive to size, rotation and position. However, it is not invariant to mirroring, so for objects which do not have symmetry with respect to an axis, two templates need to be used (see Figure 2b-c).

#### Image processing

Image data is converted into shape information by applying a consecutive set of image processing functions:

*Noise reduction* can be performed by applying Gaussian or median filtering.

**Figure 3:**
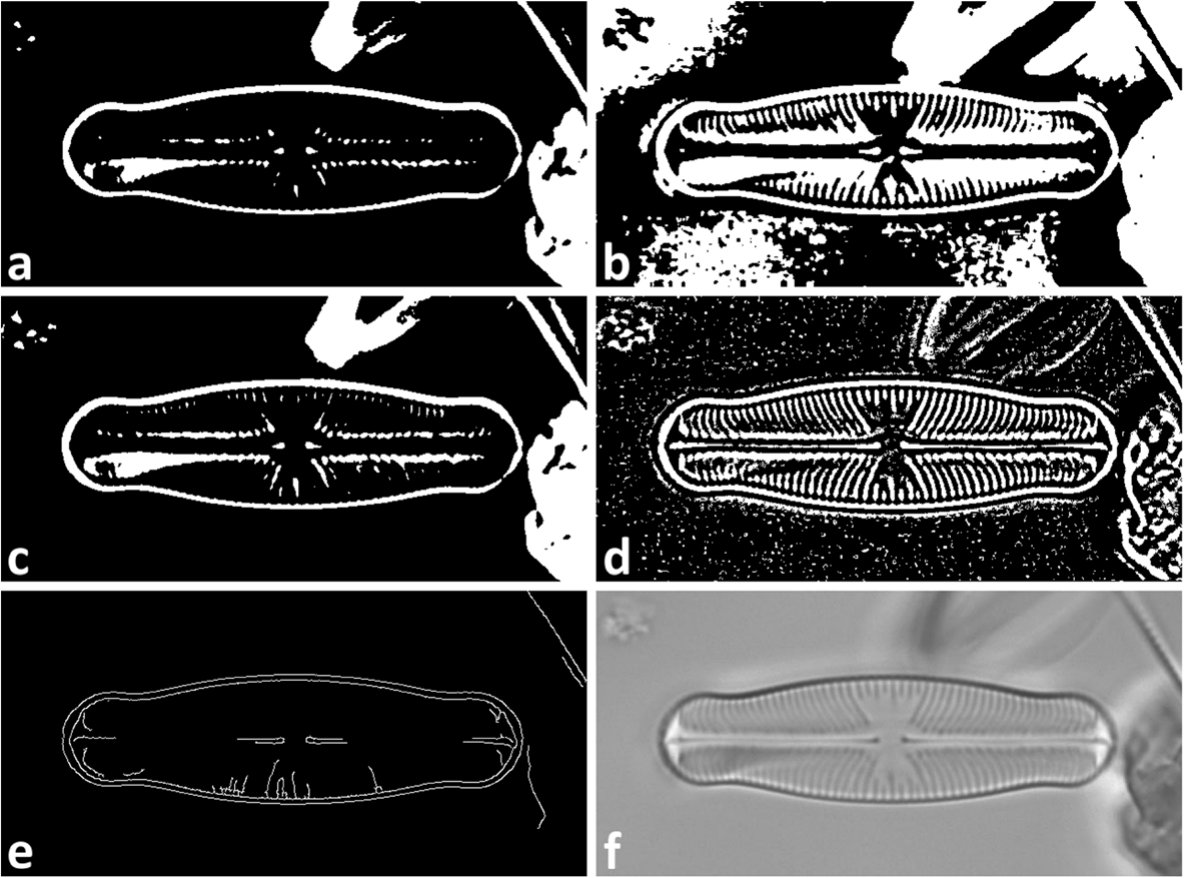
**Results of different segmentation procedures. a)** Otsu’s thresholding, **b)** Otsu’s thresholding combined with histogram equalization, **c)** robust automated threshold selector (RATS), **d)** adaptive thresholding, **e)** Canny edge detector, **f)** original image data. For each object (white) only the outer contours are analyzed subsequently.

**Figure 4:**
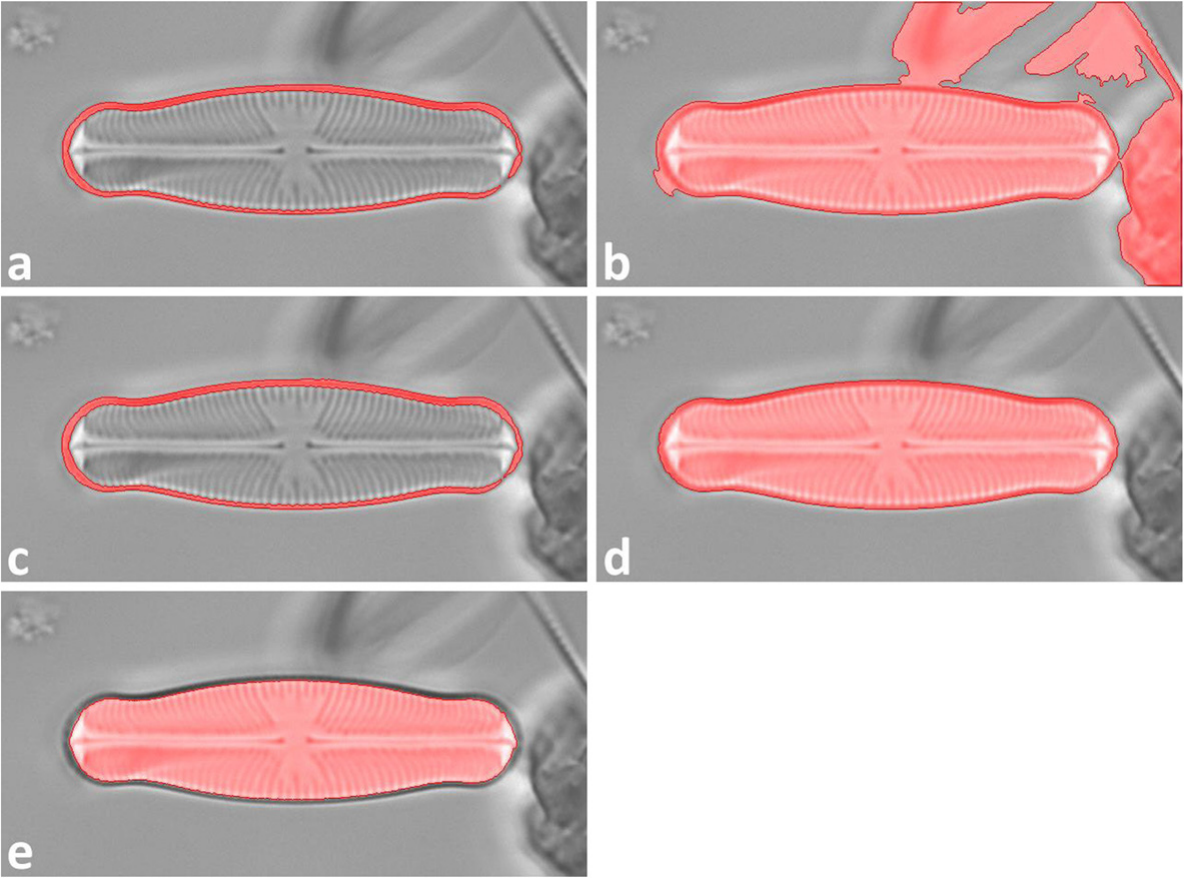
**Multiple shapes (highlighted red) detected for a diatom valve according to different segmentation procedures (compare to Figure**3**). a)** Otsu’s thresholding, **b)** Otsu’s thresholding combined with histogram equalization, **c)** robust automated threshold selector (RATS), **d)** adaptive thresholding, **e)** Canny edge detector. Only the result matching best to one of the templates (according to elliptic Fourier analysis, see below under "Shape identification") is taken for analysis.

*Image segmentation* separates objects from image background by using up to five different procedures (see Figure 3). Segmentation algorithms implemented are Otsu’s thresholding [45], Canny edge detector [46], robust automated threshold selector (RATS) [47] and adaptive thresholding [48], p. 138 ff., where Otsu’s thresholding can additionally be combined with histogram equalization [48], p. 186 ff. for analyzing images with poor contrast. Whilst for most segmentation procedures a single set of parameters is provided, RATS can be applied running a whole range of sigma values as a kind of "brute force" approach for trying to successfully segment even difficult data. Since only the outer contour of each object is analyzed, segmentation errors within the object’s interior are negligible.All segmentation procedures can be applied simultaneously. This allows for an increased yield of detected objects, since each procedure presents its own advantages and disadvantages, depending on the image data quality, but this approach can generate manifold results for a single object (see Figure 4). To prevent multiple detection, for each object only the one result will be taken into consideration, which produces the best matching value for any template (according to elliptic Fourier analysis, see below under "Shape identification"). Two shapes are considered as belonging to the same object if the centroid of one shape lies within the area of the other.

*Shape detection* is accomplished by following each object outline using an algorithm by Sklansky [49]. The outer object contour is the starting point for subsequent analysis steps.

#### Shape processing and analysis

Shapes derived from image processing might be flawed due to segmentation problems or overlapping objects, and they can depict anything from objects of interest to debris and foreign particles. To increase the yield of usable results and to sort out irrelevant data, shapes can be optimized and are evaluated according to their chance of depicting a relevant object.

**Figure 5:**
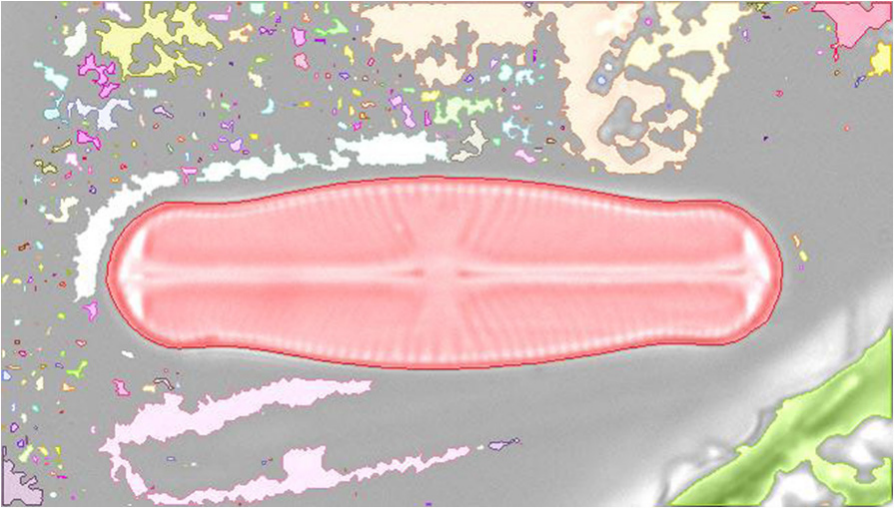
**Shapes detected after segmentation (highlighted in different colors).** Most of them do not depict relevant objects. Only the shape of the diatom valve will pass validation, other objects are too small or too close to the image border and hence are excluded from further analysis.

*Shape validation* reduces the amount of data to be analyzed to speed up the analysis processes. Each image’s segmentation can result in hundreds or even thousands of separate objects, with most of them usually not depicting relevant ones (see Figure 5). Objects will be rejected if their size is outside a user defined range, or if they are within close proximity to the image border, where the chance is high that they were truncated by the camera’s field of view.

**Figure 6:**
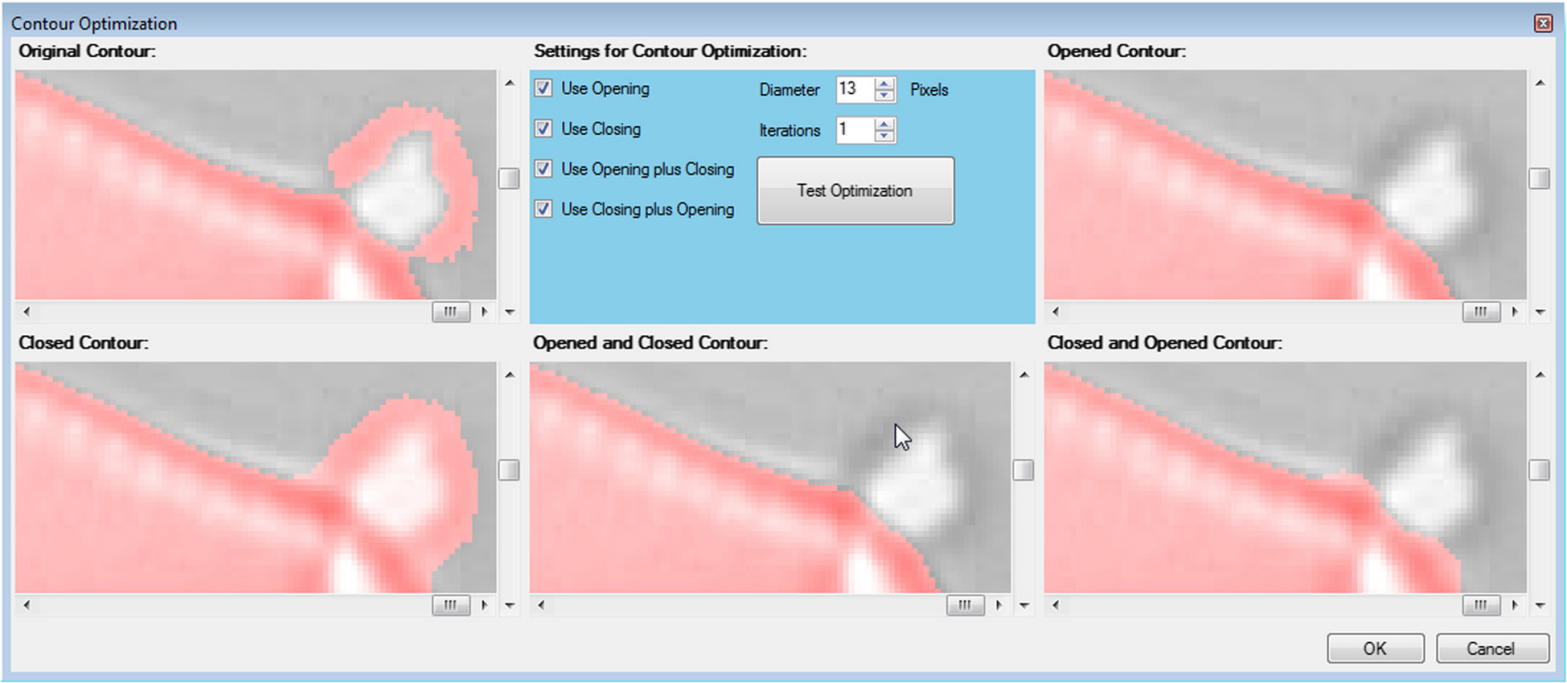
**Effects of contour optimization, shapes are highlighted in red.** The bulge of the original contour (see top left) can be eliminated successfully by applying morphological opening (see top right) or opening followed by closing (see bottom center).

*Contour optimization* can optionally be applied to increase the yield of usable results. Due to debris, overlapping structures, damages or segmentation flaws, not all objects can be segmented successfully. However, some contours can be "repaired" by applying morphological operators [50] "Opening", "Closing" and combinations of these two (see Figure 6). Small indentations and bulges are removed this way and the yield of usable results can increase significantly, but at the expense of accuracy of the derived outlines, reliability of the convexity defect measures (see below), and processing time. For each object, only the result matching best to one of the templates (see "Shape identification" below) is taken for further analysis.

*Manual rework* is an option if a shape is distorted due to segmentation flaws, but the corresponding object is essential as a valid result. SHERPA offers functions for redrawing a contour like in a painting program, for smoothing it and for applying morphological operators (see above) with individual settings to it, as well as to expand the outline to its convex hull.

**Figure 7:**
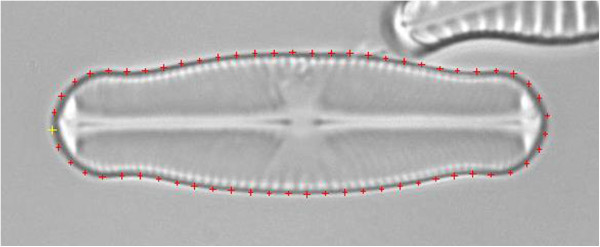
**Base points (colored crosses) used for elliptic Fourier analysis, spaced equally along the object outline.** The starting point is highlighted yellow.

*Shape identification* identifies objects by comparing their shapes with templates via elliptic Fourier analysis [51,52]. Matching is accomplished by summing up the squared differences of the normalized elliptic Fourier descriptors of object and template outline; the template having the lowest matching value is assigned to the object. The number of harmonics to be used for Fourier analysis is configurable, appropriate base points are assigned along the object perimeter at steady intervals, with the starting point being the leftmost point with respect to the major axis (see Figure 7).

#### Rating and ranking

The assignment of template and object can be incorrect either because no matching template is available, or because the object shape is distorted due to imperfect segmentation. To estimate the chance of a shape to represent a relevant object, two groups of criteria are evaluated. The first type of criteria judges the quality of shape identification plus some object features (see "Matching and quality indicators" below and Table 2), whereas the second type provides information about contour convexity (see "Convexity defect measures" below and Table 3). The user can define cut-off values for each criterion. Results are ranked by the number of criteria they fulfill. Appropriate cut-off values will depend on a number of factors, including types of objects of interest and representativeness of the template set. A guide on how to achieve appropriate settings is provided along with SHERPA’s documentation.

**Table 2:** Matching and quality indicators used for ranking

EFDIs Match with Template	Matching between elliptic Fourier descriptor invariants (EFDIs) of object and template shape [51,52].
Hu Match for EFDIs Template	Matching between the Hu invariants [55] of the object and the template which matches best according to EFDIs.
Optimization Method	Morphological Operator used to improve the object contour. If an optimization was applied to derive a shape, its ranking is degraded, because the resulting outline might be inaccurate.
Standard Deviation of inner 50%	Standard deviation of the gray level distribution within the object boundaries. Only the inner 50% of the area are analyzed. This way, diatom valves, normally containing striae/costae/areolae, can be distinguished from empty girdle bands which can produce good outline matching but have a homogenous interior.
Width/Height Ratio	Ratio between object width and height. Usually objects of a certain type have a ratio within a certain range.
Contour Smoothness	Estimation of the object contour smoothness. The actual object outline usually is quite smooth, especially for diatom valves, whilst contours distorted by segmentation inaccuracies or failures usually are rough. The ratio between the outline perimeter and that of the outline smoothed by a Gaussian filter provides information about the contour smoothness.
Formfactor	Heuristic descriptor "formfactor" [56]

**Table 3:** Convexity defect measures used for ranking

**Absolute measures**	
CDF	"Convexity Defection Factor", depicts the percentaged difference between area resp. perimeter of contour and convex hull [53]
PCAF	The "Percent Concave Area Fraction" compares the areas of contour and convex hull [54].
CHMDF	For the "Convex Hull Maximum Distance Factor" each convexity defect’s maximum distance between contour and convex hull is calculated. For distances larger than pixelwidth the squares of the distances are summed up to the CHMDF [53].
**Relative measures**	
CDF-Match	Ratio of CDF of object and template
PCAF-Match	Ratio of PCAF of object and template
Compactness-Match	Ratio of heuristic descriptor "compactness" between object and template shape

Absolute measures result from the object and are judged directly by their values, relative measures result from comparing values between object and best matching template.

*Matching and quality indicators* rate the matching between shape and template and some properties which help to distinguish objects of interest from irrelevant ones, like e.g. width/height-ratio and standard deviation of the texture gray levels within the central part of the object (see Table 2).

*Convexity defect measures* (CDMs) are calculated based on differences of area and/or perimeter between a contour and its convex hull, the latter being the smallest area which encloses the contour without containing any concave parts.

**Figure 8:**
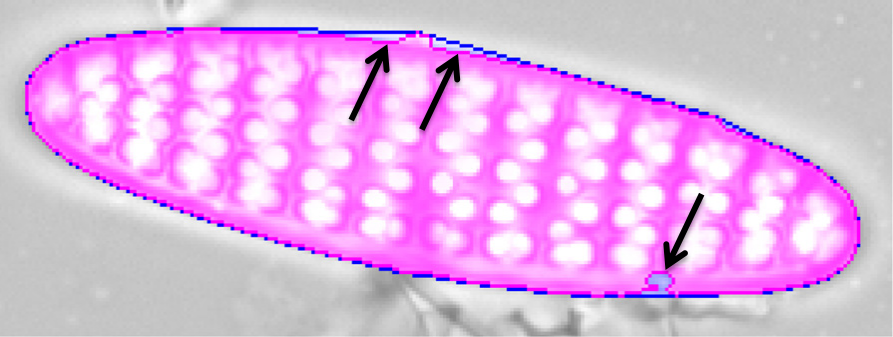
**Typical convexity defects.** The object area is highlighted in purple, its convex hull in blue. Black arrows depict significant convexity defects caused by segmentation faults, resulting in indentions resp. bulges of the contour outline.

If only convex shapes are of interest, these measures (see Table 3, "Absolute measures") are excellent features to decide about segmentation quality. This is because for convex shapes, even small indentations or bulges caused by erroneous segmentation will produce noticeable concave parts within the outline (see Figure 8), which significantly increase the CDMs. When enabling the setting "Force Convexity" in SHERPA, only absolute values of the object’s CDMs are evaluated, and only convex templates are taken into consideration. When doing so, most segmentation problems are detected clearly, and segmentation quality can be judged quite precisely based on absolute values of the convexity defect measures.

This approach will not work for objects which naturally contain concave parts. If the data contains convex as well as concave objects, SHERPA’s feature "Use Convexity" can be activated. In this case, only if the best matching template is convex, CDMs are evaluated by their absolute values derived from the respective object shape (like when using "Force Convexity"). If the best matching template is concave, some CDMs plus the heuristic descriptor "compactness" [56] of the object will be compared to those derived from the best matching template (see Table 3, "Relative measures").

When the set of objects to be detected contains both convex and concave outlines and convexity analysis is employed (i.e. "Use Convexity" or "Force Convexity" is enabled), the template set should be composed with special care. The situation to be avoided is that the best match of a concave object becomes a convex template, which can happen if no proper concave template is provided. In this case, the object convexity will be judged by absolute values even though it is concave, which will result in a failure of convexity defect measures and hence in a poor ranking.

If neither "Use Convexity" nor "Force Convexity" are activated, only a relative comparison of some CDMs between object and template plus an evaluation of the form factor takes place, regardless if the best matching template is convex or concave. The object’s CDMs are not judged directly. This is usually a good choice if it is not known in advance if all relevant objects are convex and/or there is no extensive library of templates yet.

It should be noted that detection of segmentation flaws is much less accurate when an object’s convexity defect measures are compared to those of the template instead of being judged by their absolute values. So if only convex objects are of interest, choosing "Force Convexity" will provide a more precise ranking and might save some manual reviewing.

*Heuristic descriptors* rectangularity [18], ellipticity [24], triangularity [24], roundness [56] and convexity [56,57] are calculated for exporting but not evaluated by SHERPA.

#### Review, rework and selection of results

**Figure 9:**
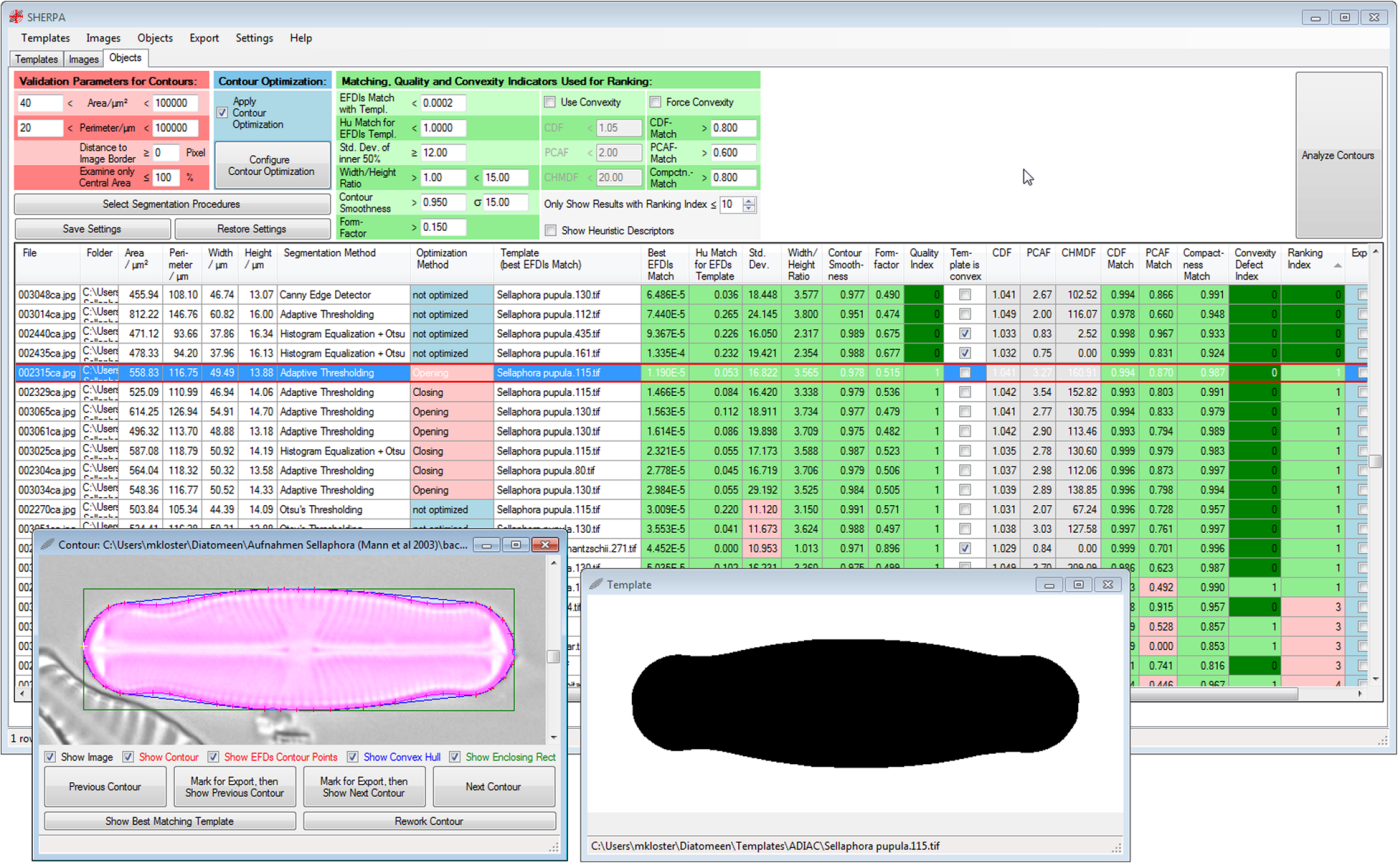
**Screenshot of SHERPA.** Analysis settings and results (background), a single result (bottom left, detected object highlighted in purple) and its best matching template (bottom center) are displayed.

Analysis results can be reviewed for verification and for selecting data to be exported in a comfortable manner (see Figure 9). For each object passing validation (see above under "Shape processing and analysis"), the path to the original image file the object was found in, the name of the segmentation method, the path to the best matching template file, values of basic morphometric variables (e.g. width, height), values of quality and convexity defect measures, and ranking are displayed. Objects can be displayed, along with their detected outlines, their enclosing convex hull, the points used for elliptic Fourier analysis as well as their best matching templates. Shapes containing segmentation errors can be reworked manually to increase the yield of usable results. Quality indicators, rankings and morphometric variables are updated after manual reworking.

#### Data export

Selected results can be exported to a set of CSV and TIFF files for further morphometric analysis using tools like e.g. "R" [37]. Results can be exported to a table containing all the information displayed by SHERPA, plus some additional morphometric values (see Table 4). All relevant settings of SHERPA used to create these results are stored into a separate file. Optionally, the image data cropped to the object region, the coordinates of the object outline, the coordinates of the outline points used for elliptic Fourier analysis, and the resulting descriptors can be exported to separate files for each result. Detailed information on all features is included in the manual and the "Technical Details" document linked within SHERPA’s help menu.

**Table 4:** Exportable features

**Name of feature**	**Description**
Source Image	Path to raw image data file
Area	Object area
Perimeter	Object perimeter
Width	Object width (along major axis)
Height	Object height (perpendicular to major axis)
Rotation Angle	Rotation angle of the major axis
Segmentation Method	Segmentation method used to derive the object shape
Optimization Method	Optimization method applied to the object shape
Best Template (EFDIs)	Path to the best matching template (according to matching of elliptic Fourier descriptor invariants)
Template Difference (EFDIs)	Value for matching of elliptic Fourier descriptor invariants between object and best matching template
Hu-match for best EFDIs-Template	Value of matching of Hu invariants between object shape and best matching template
Standard Deviation	Standard deviation of texture gray levels within the inner 50% of the object boundaries
Width/Height-Ratio	Aspect ratio of the object shape
Smoothed Perimeter Ratio	Ratio between the perimeters of the smoothed and the original contour; smoothing is performed by Gaussian filtering of the contour coordinates.
Quality Index	Number of fulfilled quality indicators
Template is convex	Indicator showing if the best matching template is convex
Convexity is used	Indicator showing if convexity was judged directly to calculate convexity indicators (use of absolute convexity measures)
Rectangularity	Heuristic descriptor
Compactness	Heuristic descriptor
Ellipticity	Heuristic descriptor
Triangularity	Heuristic descriptor
Roundness	Heuristic descriptor
Convexity by perimeter	Heuristic descriptor
Convexity by area	Heuristic descriptor
Formfactor	Heuristic descriptor
CDF	Convexity defect measure
PCAF	Convexity defect measure
CHMDF	Convexity defect measure
CDF-Match	Ratio of CDF between object and template
PCAF-Match	Ratio of PCAF between object and template
Compactness-Match	Ratio of heuristic descriptor "formfactor" between object and template
Convexity Defect Index	Number of fulfilled absolute or relative convexity indicators
Ranking Index	Ranking for object shape, i.e. estimation of quality and relevance of result
Contour Image	Name of the file containing the image data cropped to the object area
Contour Image top left Corner	Coordinates of the top left corner of the cropped object image with respect to the raw data
Image Moments (mu)	Image moments of the object shape
Hu Invariants (Hu)	Hu-Invariants of the object shape

## Results and discussion

For the following analyses, bright field micrographs of valves of different diatom species and from different sources were analyzed. All results were produced without manually reworking or resorting detected shapes, relying solely on SHERPA’s automated functions for segmentation, contour optimization and result ranking.

### Templates

To facilitate use of SHERPA for generic diatom recognition and analysis, we prepared a library covering a wide range of diatom outline shapes, containing about 450 templates. This compilation is mainly based on the outline shape classification scheme and accompanying diagrams from Barber & Haworth [58], *Fragilariopsis* data sets from a surface sediment sample [59], and upon the extensive ADIAC diatom image database available online [44], although the ADIAC data is not fully covered by the current template library. For the latter two, SHERPA was used for image segmentation to detect shapes previously not represented in the template set: Shapes with a poor template matching value were screened manually. If they were depicting relevant valves and segmentation quality was satisfactory, they were converted into additional templates employing the built-in functions of SHERPA. Because diatom shapes vary widely among taxa, as well as during the life cycle of even a single taxon, it is crucial to check the presence of a representative set of templates for taxa of interest when using SHERPA for analyzing a particular type of diatom samples.

#### *Sellaphora* data as example for identification accuracy

To demonstrate the usability of SHERPA, we analyzed a set of images from one of the classical model taxa of diatom microdiversity, the *Sellaphora pupula* (Kützing) Mereschkowsky complex s.l. *S. pupula* has been known as a morphologically highly variable diatom species during most of the 20^th^ century. However, Mann and colleagues demonstrated in a series of papers (cumulating in [7]) that sympatric demes of this diatom "species" formed reproductively isolated groups, that could also be diagnosed using molecular markers and also differed in minute morphological/morphometric features, including (but not limited to) minor differences in their valve outlines. In their 2004 investigation [7], Mann et al. used Legendre-polynomials and contour segment analysis for comparing outline morphology of six *S. pupula* demes (since that study, also formally recognized as distinct species). They made the images upon which the analyses were based publicly available [60], which we used in this analysis.

All five segmentation methods plus contour optimization were applied to analyze a total of 383 micrographs focused on the outlines of *Sellaphora* valves (see Table 5). Most of the valves were clearly isolated, without overlapping structures and only little amount of debris, so this might not be a typical data set, but serves as an example on how specific the identification process works. Since contours of *S. pupula* contain concave parts, convexity was not taken into account for judging segmentation quality directly (i.e. neither "Use convexity" nor "Force convexity" were activated in SHERPA).

**Table 5:** **Results analyzing 383 images**[60]**depicting *Sellaphora *** **valves (plus one centric diatom)**

	**Identified as *Sellaphora pupula***	**Identified as other**^**1)**^
Ranking 0	318	4
Ranking 1	25	7
Ranking 2	2	1

^1)^One centric diatom was present in the data set, the other valves identified as being not *Sellaphora* have a similar shape and therefore cannot be distinguished when using the large template set.

All five segmentation methods were used (RATS with σ range 1 to 11) and contour optimization was applied.

**Figure 10:**
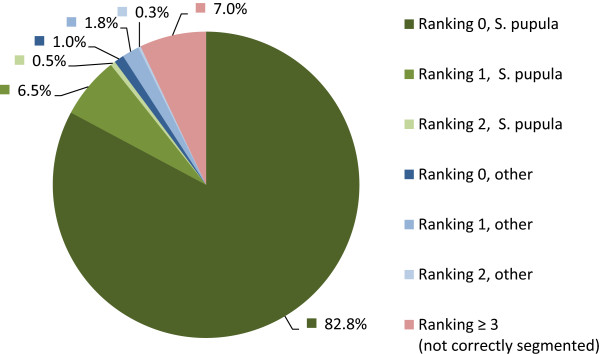
**Percentage of different rankings and identifications for the *Sellaphora *data set (compare Table**5**).** About 93% of the valves were segmented successfully (green and blue), about 90% were identified correctly as *S. pupula* (green), about 7% were not segmented successfully (red).

Considering only results of ranking 0 to 2, which usually is the range for objects without significant segmentation flaws and good coverage by templates, 357 (93%) of the valves contained in the data set were successfully segmented (see Figure 10). When using the comprehensive template library, most of the results were assigned correctly to one of the 18 *Sellaphora pupula* templates derived from the ADIAC dataset (no template was created from the *Sellaphora* data set itself). Only about 3% of the results were assigned to templates of other species, which had shapes very similar to *S. pupula*. One centric diatom was actually present in the data and correctly identified as a disc-shaped type, clearly distinct from the others. When using only the 18 *Sellaphora pupula* templates instead of the whole template library, the yield was identical (apart from the single centric diatom), with all valves correctly identified.

Results having a ranking above 2 are not listed, because they were caused by partly unfocused outlines, overlapping objects or debris and would have needed manual inspection and reworking.

#### *Fragilariopsis* data as example for segmentation quality

As a typical data set, 773 micrographs originating from sediment core PS1768-8 [59] and mainly showing *Fragilariopsis kerguelensis,* plus broken valves, debris and overlapping objects, were analyzed. The data was obtained using a Metafer slide scanning system (Metasystems, Altlussheim, Germany), applying the implemented autofocus and stacking functions. Because not all valves were lying parallel to the focal plane, outlines were partly out of focus or blurred despite of stacking. Since the outline of *F. kerguelensis* is completely convex, SHERPA’s "Force Convexity" feature was used to improve judging of segmentation quality.

Again, the full template set covering a broad range of diatom species was used. Although *Fragilariopsis* valves were mostly identified correctly, some were assigned to templates of other similarly shaped species, and some correctly identified valves of other species were present. Undamaged valves could successfully be distinguished from artifacts like broken ones or debris. In some cases, objects like girdle bands or spherical structures were identified as relevant valves (usually at a ranking index 2 or worse), because of their shape similar to those of other diatom species in the template library. This problem can be overcome by using only *Fragilariopsis* templates.

**Figure 11:**
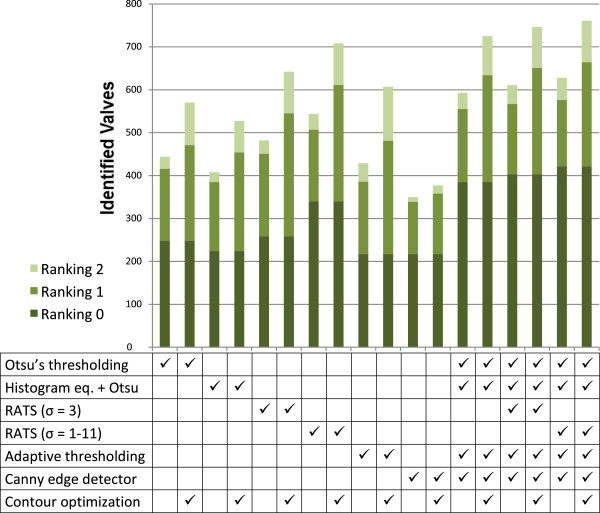
**Results for *Fragilariopsis *data for different combinations of segmentations methods and contour optimization (compare Table**6**).** The more methods are combined, the higher is the yield.

**Figure 12:**
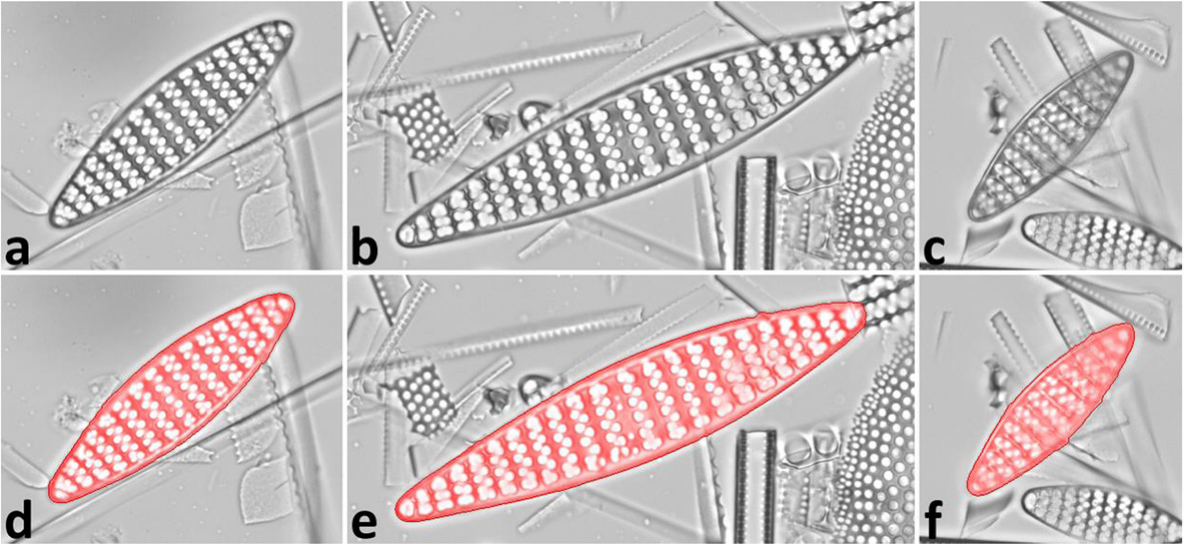
**Successful segmentation in the presence of debris and overlapping objects. a)** – **c)** Micrographs of *Fragilariopsis* valves, **d)** – **f)** segmented shapes (highlighted red) after contour optimization, using **d)** adaptive thresholding, **e)** Otsu’s thresholding, **f)** RATS (σ = 3.0). By application of multiple segmentation methods and contour optimization even problematic objects could be extracted, since often at least one of the methods succeeded, but partly at the expense of contour accuracy (see the small bulges in the object contours).

All segmentation methods available in SHERPA were applied separately, as well as in combination, to compare the yield of usable results (see Table 6 and Figure 11). As expected, the best yield is achieved when using all segmentation methods, employing RATS with a wide range of σ, and applying contour optimization. When combining the individual strengths of the different methods plus contour optimization, even objects which are difficult to segment can be handled successfully; although not always without contour inaccuracies (see Figure 12). Since applying the whole range of methods drastically increases the time needed for analysis, using only Otsu’s thresholding, Canny edge detector, adaptive thresholding and Otsu’s thresholding plus histogram equalization might be a practicable choice for preliminary or quick analyses.

**Table 6:** **Results for *Fragilariopsis *** **data for different combinations of segmentations methods and contour optimization**

**Otsu’s thresholding**	**Histogram equalization**	**RATS (σ = 3)**	**RATS (σ = 1-11)**	**Adaptive thresholding**	**Canny edge detector**	**Contour optimization**	**Ranking 0 total**	**Ranking 1 total**	**Ranking 2 total**^**2)**^	**Total ranking 0 to 2**
✓							248	168	28	444
✓						✓	248	223	99	570
	✓						224	161	23	408
	✓					✓	224	230	73	527
		✓					258	193	31	482
		✓				✓	258	287	97	642
			✓				340	167	37	544
			✓			✓	340	271	97	708
				✓			217	169	43	429
				✓		✓	217	264	126	607
					✓		217	122	11	350
					✓	✓	217	141	19	377
✓	✓			✓	✓		385	170	38	593
✓	✓			✓	✓	✓	385	249	91	725
✓	✓	✓		✓	✓		403	164	44	611
✓	✓	✓		✓	✓	✓	403	248	95	746
✓	✓		✓	✓	✓		421	155	52	628
✓	✓		✓	✓	✓	✓	421	243	97	761

^2)^Whilst results of ranking 0 and 1 contain nearly only correctly segmented valves of *Fragilariopsis* and a few of other species, ranking 2 also contains few results of girdle bands incorrectly identified as valves.

The more methods are combined, the higher is the yield.

### Comparison of segmentation methods

**Figure 13:**
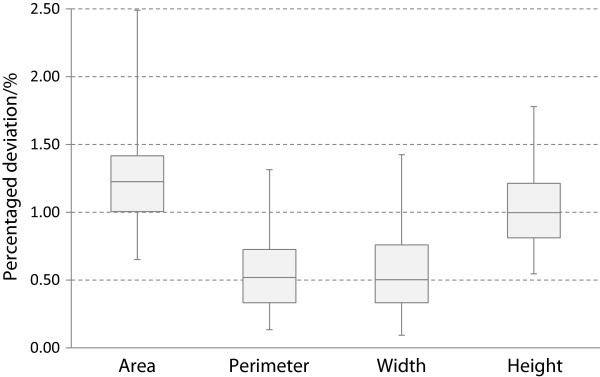
**Boxplots of percentaged deviation of features around the minimum/maximum center when using all five segmentation methods.** The deviation is about ± 1% around the center value.

88 valves of the *Fragilariopsis* data were successfully segmented by each of the five segmentation methods (RATS with σ = 3.0) without applying contour optimization. Area, perimeter, width and height obtained by the different segmentation methods were compared by calculating their percentage deviation for each of these valves. The deviations for all valves were compared (see Equation 1). This illustrates the variation of the object contours produced by the different segmentation methods, which is about ±1% around the center value between the minimum/maximum values (see Figure 13).

(1) $$\text{Percentaged deviation}=\frac{\text{MAX}-\text{MIN}}{\text{MAX}+\text{MIN}}\cdot 100\;\%$$

With *MAX* = maximum, *MIN* = minimum value for a feature (area, perimeter, etc.) when using multiple segmentation methods.

### Further analysis using R

**Figure 14:**
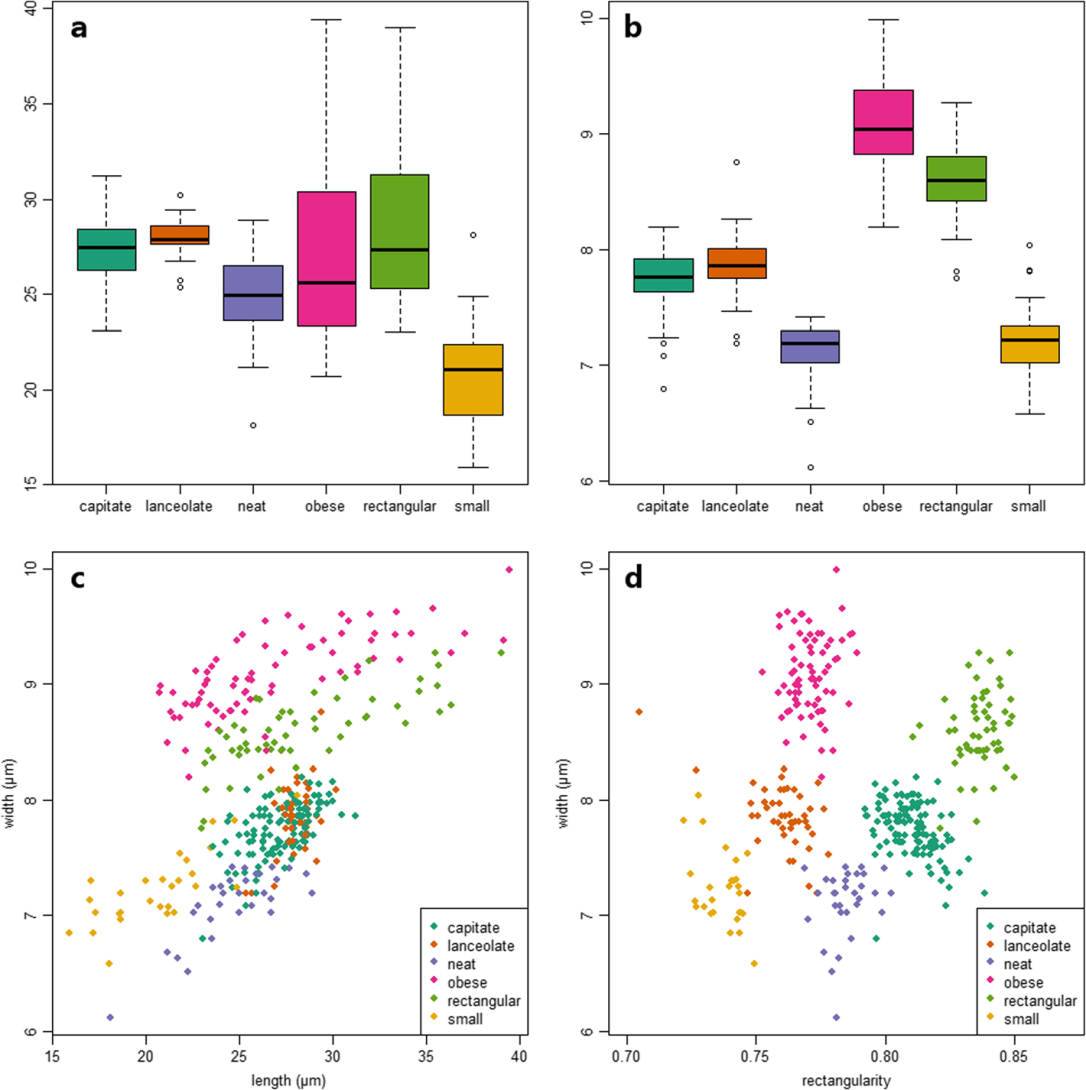
**Reproduction of plots from Mann et al. **[7]**using the same variables. a)** valve length, **b)** valve width, **c)** valve width vs. length, **d)** valve width vs. rectangularity, corresponding to Figures 5, 6, 10 and 14 from Mann et al. [7]. In the box plots in **a)** and **b)**, the thick horizontal lines represent the medians; the boxes range from the first to the third quartile; and whiskers +/- 1.58 times the interquartile range. Individual values outside these ranges are displayed as circles.

As a benchmark experiment, and to illustrate how data exported by SHERPA can be used in further analyses, we imported both the classical morphometric features and the elliptic Fourier descriptors (EFDs) calculated by SHERPA for the 356 *Sellaphora* valves from the first above described experiment into the open source statistical data analysis environment R [37]. In R, we reproduced those plots from Mann et al. [7] for which features used were captured by SHERPA (see Figure 14; besides outline features, Mann et al. also measured a number of features characterizing striae density, orientation and the terminal bars which are not captured by SHERPA).The plots correspond to Figures 5, 6, 10 and 14 from Mann et al., based on valve length, width and rectangularity. These figures rather accurately correspond to those in the original publication, with the exception of a single "lanceolate" valve with an extremely low rectangularity value of 0.705: such a low value does not appear in the original publication and it is also extremely low when compared with the other values exported from SHERPA. This outlier reflects a segmentation problem caused by a shadow overlapping the valve outline which can easily be fixed using the "Manual rework" feature of SHERPA, resulting in a rectangularity value of 0.757 which hardly differs from the value given for the same valve by Mann et al. (0.760). In order to illustrate the accuracy of the methods when applied in a fully unsupervised manner, we opted to keep the original value for Figure 14a) and for the following classification exercise. When applying a cross-validation linear discriminant analysis based on classical morphometric features extracted by SHERPA (randomly selected 50% of objects used to train the model, the remaining 50% is then classified against it, in 100 iterations), classification accuracies of the six demes (species) range from 98.9% to 100% (median: 100%).

**Figure 15:**
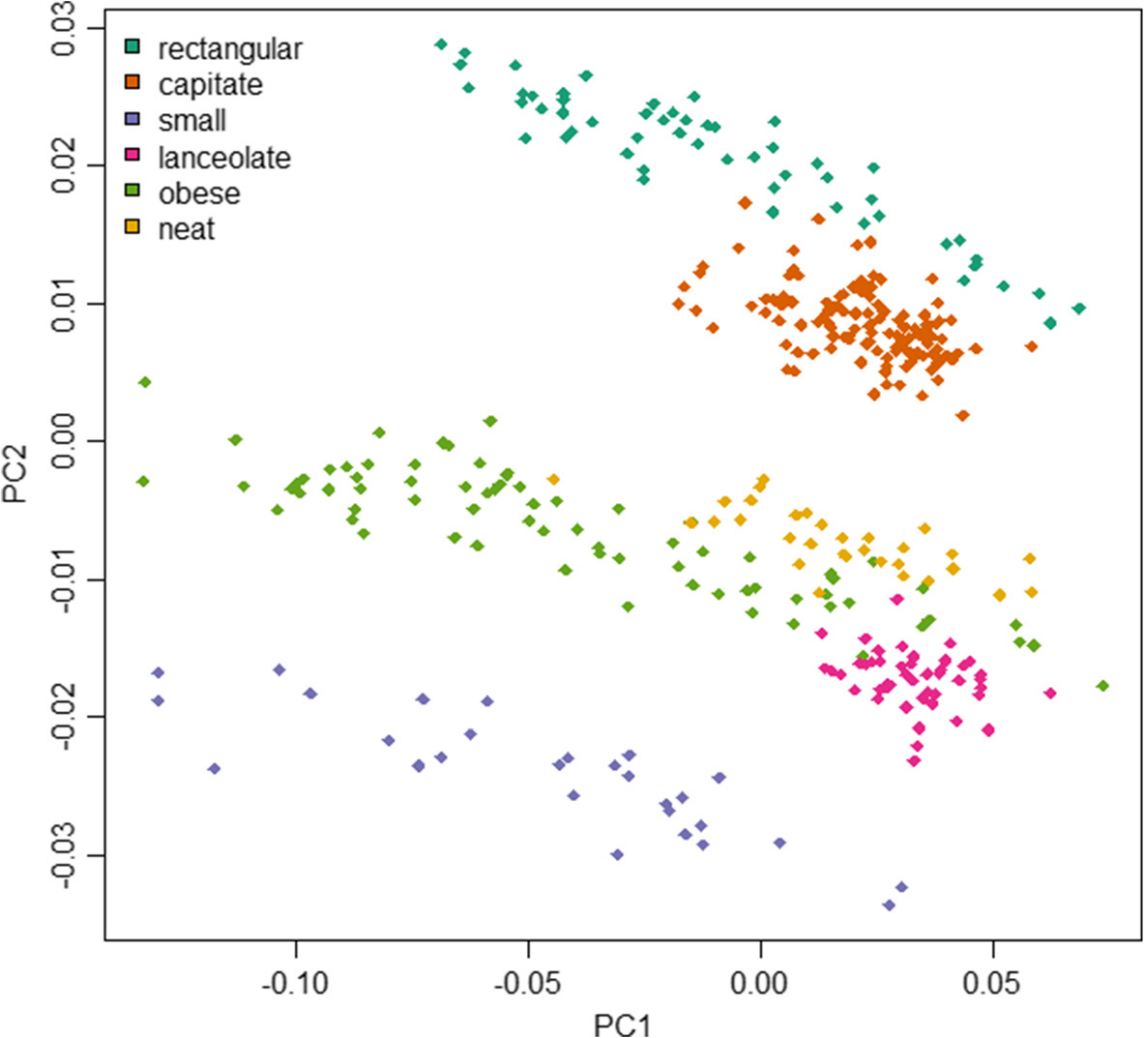
**Principal component analysis of elliptic Fourier descriptor invariants for the *Sellaphora *data set.** EFDIs have a comparable discriminatory power to the Legendre polynomials used by Mann et al. [7], differentiating the three main shape groups but not the individual demes/species within each shape group.

EFDIs performed less well in linear discriminant analysis (77.5 - 92.7% accuracy, median: 88.2%, in an identical cross-validation, see Figure 15), but the classical morphometric features still demonstrate that the set of features extracted by SHERPA provides a robust basis for downstream outline-based classification, especially when considering the small differences in outline shapes among the *Sellaphora* groups.

### Future development

Besides improving performance, the next steps in SHERPA’s development will concern the analysis of texture und structural features to improve versatility and identification specificity.

## Conclusions

SHERPA provides a useful tool for diatom identification and morphometrics, enabling mass screenings, since it greatly reduces the amount of work needed to be performed by human interaction. Manual revision required for best results can be accomplished in a quick and effective manner, supported by a ranking based on matching and quality indicators.

The degree of identification reliability reflects both the range of templates used and the diversity present in the analyzed samples. In spite of depending solely on outline shape, good identification accuracy can be reached using customized template sets. Combining multiple segmentation methods improves the identification rate without significantly impairing result accuracy, and, combined with contour optimization, even objects showing segmentation artifacts can be analyzed successfully. For convex shapes, convexity defect measures provide an effective way to judge segmentation quality, hence allowing identification of flawed object outlines.

The approach of restricting SHERPA to the identification of relevant objects and the calculation of their morphometric features enables an adaptation to specific problems/target taxa. Downstream analyzes or classification can be performed using widely available commercial or free statistical software tools, e.g. "R".

## Availability and requirements

**Project name:** SHERPA.

**Project home page:**http://www.awi.de/sherpa.

**Operating system(s):** Windows7 64 Bit (32 Bit version available).

**Programming language:** C#.

**Other requirements:** .NET 4.0.

**License:** Freeware, royalty-free, non-exclusive.

**Any restrictions to use by non-academics:** none.

## Abbreviations

SHERPA: Tool for "Shape recognition, processing and analysis"; CDMs: Convexity defect measures; EFDs: Elliptic Fourier descriptors; EFDIs: Elliptic Fourier descriptor invariants; CDF: "Convexity defection factor", a convexity defect measure; PCAF: "Percent concave area fraction", a convexity defect measure; CHMDF: "Convex hull maximum distance factor", a convexity defect measure.

## Competing interests

The authors declare that they have no competing interests.

## Authors’ contributions

MK developed SHERPA and its image processing workflow, performed the data analyses, and is the main author of this paper. GK mentored the beginning steps of SHERPA (at this point called "DiatoMorphoTo" and part of MK’s master thesis) and revised the manuscript. BB strongly contributed to the morphometric aspects of SHERPA as well as this paper, took care of the "R" part and was the main information source on diatom taxonomy. All authors read and approved the final manuscript.

## Authors’ information

MK started developing SHERPA as part of his master thesis (at that time called "DiatoMorphoTo") at the HSEL, supervised by GK and in collaboration with BB. Since graduation he works at the Friedrich Hustedt Diatom Study Centre, AWI, under supervision of BB to develop SHERPA. He mainly works at the interface between biology and informatics, focusing on image processing, data visualization and automation.

GK is a professor in bioinformatics and has been working for more than 15 years in the areas of genome analysis, microscopy, image processing, image interpretation and development of bioinformatics methods for genome and proteome analysis. His recent works regard high performance reconstruction of structures from high resolution image stacks of extensive microscopic objects, and limited three-dimensional reconstruction from stereoscopic images of biological tissues and organisms. GK is also the author of E.L.M.I. (Expert System for Light Microscopy).

BB is a diatomist / bioinformaticist, curator of the Hustedt Diatom Study Centre. His research currently focuses on taxonomy, biogeography and morphometrics of Antarctic diatoms.
